# The multi-grip and standard myoelectric hand prosthesis compared: does the multi-grip hand live up to its promise?

**DOI:** 10.1186/s12984-023-01131-w

**Published:** 2023-02-15

**Authors:** Nienke Kerver, Verena Schuurmans, Corry K. van der Sluis, Raoul M. Bongers

**Affiliations:** 1 Department of Rehabilitation Medicine, University of Groningen, University Medical Center Groningen, Groningen, The Netherlands; 2 Department of Human Movement Sciences, University of Groningen, University Medical Center Groningen, Groningen, The Netherlands; 3 Department of Rehabilitation Medicine, University of Groningen, University Medical Center Groningen, Groningen, The Netherlands; 4 Department of Human Movement Sciences, University of Groningen, University Medical Center Groningen, Groningen, The Netherlands

**Keywords:** Upper extremity, Amputation, Prostheses, Compensation, Functionality, User experience, International Classification of Functioning, Disability, and Health, Artificial limbs

## Abstract

**Background:**

Multi-grip myoelectric hand prostheses (MHPs), with five movable and jointed fingers, have been developed to increase functionality. However, literature comparing MHPs with standard myoelectric hand prostheses (SHPs) is limited and inconclusive. To establish whether MHPs increase functionality, we compared MHPs with SHPs on all categories of the International Classification of Functioning, Disability, and Health-model (ICF-model).

**Methods:**

MHP users (N = 14, 64.3% male, mean age = 48.6 years) performed physical measurements (i.e., Refined Clothespin Relocation Test (RCRT), Tray-test, Box and Blocks Test, Southampton Hand Assessment Procedure) with their MHP and an SHP to compare the joint angle coordination and functionality related to the ICF-categories ‘Body Function’ and ‘Activities’ (within-group comparisons). SHP users (N = 19, 68.4% male, mean age = 58.1 years) and MHP users completed questionnaires/scales (i.e., Orthotics and Prosthetics Users’ Survey—The Upper Extremity Functional Status Survey /OPUS–UEFS, Trinity Amputation and Prosthesis Experience Scales for upper extremity/TAPES-Upper, Research and Development-36/RAND-36, EQ-5D-5L, visual analogue scale/VAS, the Dutch version of the Quebec User Evaluation of Satisfaction with assistive technology/D-Quest, patient-reported outcome measure to assess the preferred usage features of upper limb prostheses/PUF-ULP) to compare user experiences and quality of life in the ICF-categories ‘Activities’, ‘Participation’, and ‘Environmental Factors’ (between-group comparisons).

**Results:**

‘Body Function’ and ‘Activities’: nearly all users of MHPs had similar joint angle coordination patterns with an MHP as when they used an SHP. The RCRT in the upward direction was performed slower in the MHP condition compared to the SHP condition. No other differences in functionality were found. ‘Participation’: MHP users had a lower EQ-5D-5L utility score; experienced more pain or limitations due to pain (i.e., measured with the RAND-36). ‘Environmental Factors’: MHPs scored better than SHPs on the VAS-item holding/shaking hands. The SHP scored better than the MHP on five VAS-items (i.e., noise, grip force, vulnerability, putting clothes on, physical effort to control) and the PUF-ULP.

**Conclusion:**

MHPs did not show relevant differences in outcomes compared to SHPs on any of the ICF-categories. This underlines the importance of carefully considering whether the MHP is the most suitable option for an individual taking into account the additional costs of MHPs.

**Supplementary Information:**

The online version contains supplementary material available at 10.1186/s12984-023-01131-w.

## Introduction

Myoelectric hand prostheses can replace a human hand after amputation or a congenital transradial deficiency with the goal to restore cosmetic appearance and function [1]. A myoelectric prosthesis is controlled by muscle signals in the residual limb recorded with electromyography electrodes. Direct control is the most used, in which an electrode is placed on the skin above each of two antagonistic muscles [2]. Usually, activating the extensor muscles of the wrist will open the hand, while activating the flexor muscles will close the hand. Triggers, such as double pulses or co-contractions, can be used to switch grips [3]. A standard myoelectric hand prosthesis (SHP), such as the Myohand Variplus Speed (Ottobock; Duderstadt, Germany) or Motion Control Hand (Fillauer, USA), has a movable thumb, index finger, and middle finger that can open and close in only one grip, the tripod grip. A multi-grip myoelectric hand prosthesis (MHP), such as the i-Limb Quantum/Ultra (Össur; Reykjavík, Iceland) and the BeBionic (Ottobock; Duderstadt, Germany), has five movable and jointed fingers making it possible to produce multiple grips (e.g., pointing index finger, pinch grip, key grip). As more grips are available with an MHP, an increased dexterity is a benefit that users often mention [4–6]. However, numerous disadvantages of MHPs have been mentioned as well, such as fragility, costs, and difficult and tedious control [4, 5]. The triggers required to switch grips are often experienced as time-consuming and cognitively demanding [5]. Considering these mixed opinions about the MHP, the question arises whether the MHP and the simpler SHP differ in functionality and user experiences. Even though MHPs are available for already 15 years, research conducted on these differences is limited and inconclusive, which might be due to small sample sizes and the lack of diversity (e.g., sex and age) in the test groups [7–10]. Therefore, we aimed to compare MHPs and SHPs on the most relevant domains regarding prosthesis use, for which we applied the International Classification of Functioning, Disability, and Health-model (ICF-model) [11, 12]. The ICF-model characterizes someone’s health and functioning based on Body Functions, Activities, and Participation. Prosthesis use can influence these factors and is categorized within ‘Environmental factors’.

The category ‘Body Function’ encompasses the functioning of the body, such as the movements produced by the muscles, and how joints are coordinated [13]. Most of the previous studies focus on measuring task completion or movement time, implicitly assuming that those measures reflect joint coordination [13]. In some cases, a prosthesis user can perform a movement as fast and accurately as an able-bodied person. However, the joint coordination pattern may differ considerably [7, 14–16], since a prosthesis user often uses more proximal joints to compensate for the loss of the distal degrees of freedom, such as the wrist [16]. Studies that focussed on differences in joint coordination (i.e., compensatory movements) during prosthesis use, found that prosthesis users showed an increase in range of joint motion (RoM) of trunk and shoulder angles compared to able-bodied individuals [14–16]. While these compensatory movements lead to momentarily successful completion of tasks, they may cause overuse complaints in the long term. Previous studies suggest that an MHP might decrease these compensation movements [7, 14, 16], but as far as we know, no studies actually compared the joint coordination of the MHP and the SHP.

The category ‘Activities’ revolves around the functioning of the individual in their environment. To assess performance in this category, it is established which tasks can be completed with the prosthesis and how fast and accurate these can be performed. Frequently used measurement instruments are the Southampton Hand Assessment Procedure (SHAP), Box and Blocks Test (BBT) & Orthotics and Prosthetics Users’ Survey-The Upper Extremity Functional Status Survey (OPUS-UEFS). In previous research, little evidence is found about the increase of dexterity with the MHP compared to the SHP. The case report of van der Niet et al. found no significant differences on OPUS-UEFS scores between an MHP and an SHP hand [8]. Additionally, five studies showed no differences between the two hands on the SHAP and BBT [8–10, 17, 18]. Only one study stated an increase in fine motor control with an MHP [6], although it should be noted that this research focussed on the Michelangelo hand, which was excluded from the current study.

‘Participation’ focuses on the functioning of the individual in society. Questionnaires that have been used in upper limb prosthesis (ULP) users to assess participation are the Trinity Amputation and Prosthesis Experience Scales Upper extremity (TAPES-Upper), EQ-5D, and the Research and Development-12/36 (RAND-12/36). Previous studies found no differences in (components of) TAPES-upper and EQ-5D [6, 8]. Additionally, no differences were found in Veteran RAND-12 Item Health Survey scores, which is comparable to the RAND-12, between users of the MHP and the SHP [9].

‘Environmental factors’ refer to external factors, such as a prosthesis, that can influence ‘Body Functions’, ‘Activities’, and ‘Participation’. Evaluation measures that have been mentioned in the literature are the Dutch version of the Quebec User Evaluation of Satisfaction with assistive technology (D-QUEST), visual analogue scales (VAS-scores), and the electronic patient-reported outcome measure (ePROM) to assess the preferred usage features of upper limb prostheses (PUF-ULP) [19]. Two previous studies showed that users were more satisfied with the MHP than with the SHP [8, 18]. However, another study revealed that half of the MHP users switched to a different terminal device within a year [20].

As described above, there are still gaps in the existing literature on the differences between MHPs and SHPs. Therefore, this study aimed to compare the MHP and SHP on the ICF categories ‘Body function’ and ‘Activities’ (i.e., joint coordination, dexterity, prosthetic hand function) within a group of experienced MHP users. Secondly, we aimed to compare the MHP and SHP on ‘Activities’, ‘Participation’, and ‘Environmental factors’ (i.e., user experiences, satisfaction with the prosthesis, adjustment to upper limb absence (ULA) and prosthesis, quality of life) between separate groups of MHP-users and SHP-users.

## Methods

### Participants

Eligible MHP and SHP users were approached through nine rehabilitation centres and two orthopaedic workshops in the Netherlands. Additionally, interested people from our previous national survey study were approached [19] and an advertisement for participation was placed on the website of the patient association for people with limb absence in The Netherlands.

The experiment included two different test groups: one group with MHP users (group MHP) and another with SHP users (group SHP). The general eligibility criteria for both groups were (1) age ≥ 18 years; (2) ULA at transradial level or wrist disarticulation; (3) sufficient command of the Dutch language to follow instructions and to complete questionnaires/scales; (4) in a stable phase of the prosthesis provision process meaning at least six months experience with either their MHP or SHP; (5) no co-morbidities that could influence the results of this study, like neurological disorders, cognitive disorders, rheumatic diseases, and other disorders that affect arm function.

An additional eligibility criterion for the MHP group was that they were in possession of a myoelectric prosthesis with an MHP, type: i-Limb Quantum/Ultra (Touch Bionics; Livingston, United Kingdom), BeBionic (Ottobock; Duderstadt, Germany) or VINCENT (Vincent Systems, Karlsruhe, Germany). People with a Michelangelo hand (Ottobock; Duderstadt, Germany) were considered ineligible because not all fingers are motorized, nor can this hand easily be exchanged with an SHP due to a different wrist connection. Additional eligibility criteria for the SHP group were (1) in possession of an SHP, and (2) able to fill in an online questionnaire. The sample size was determined based on the research of Luchetti et al., which compared the MHP and SHP using the BBT and the SHAP [6]. G-power was used to calculate the adequate sample size [21]. With an α set at 0.05 and the statistical power at 0.8, 13 participants were needed for the current study to find a significant difference between the two prosthetic hands.

The measurements were conducted at two different rehabilitation centres in the Netherlands: University Medical Centre Groningen (UMCG; Groningen) and Libra Rehabilitation & Audiology (Eindhoven). The local Medical Ethics Review Board of the UMCG waived formal study approval (METc 2018/582). This study was carried out in compliance with the Declaration of Helsinki and the COVID-19 measures imposed by the contributing institutions and the Dutch government. Participants provided written informed consent before study entry. Participants from MHP and SHP groups received €150 and €20, respectively, as a reward for completing the study protocol. Additionally, the MHP group received an allowance for travel expenses.

### Study design and procedure

This cross-sectional study had a limited cross-over design, which consisted of two parts: (1) between-group comparison using questionnaires/scales and (2) within-group comparison based on physical measurements.

**Fig. 1:**
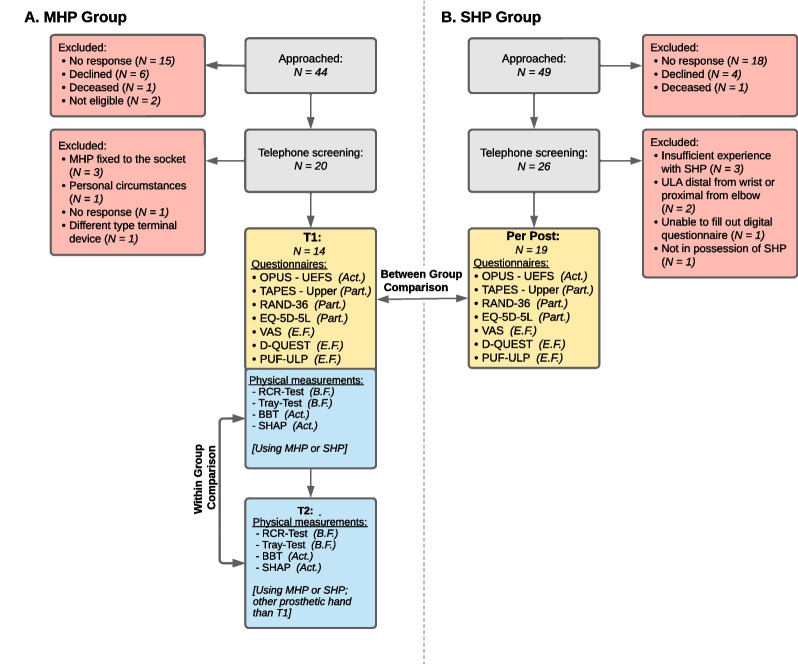
Schematic overview of the study design. At T1, for the MHP group, both the questionnaires/scales and physical measurements were conducted. At T2 only the physical measurements were performed. The red colour specifies the excluded participants. Yellow represents the between-group comparison, while blue represents the within-group comparison. *MHP *multi-grip myoelectric hand prosthesis, *SHP* standard myoelectric hand prosthesis, *ULA* upper limb absence, *ICF* International Classification of Functioning, Disability, and Health-model, *B.F.* Body Function, *Act* Activities, *Part.* Participation, *E.F.* Environmental Factors, *OPUS*-*UEFS* Orthotics and Prosthetics Users’ Survey-The Upper Extremity Functional Status Survey, *TAPES-Upper* Trinity Amputation and Prosthesis Experience Scales for upper extremity, *RAND-36* Research and Development-36, *VAS *visual analogue scales, *D-QUEST* Dutch version of the Quebec User Evaluation of Satisfaction with assistive technology, *PUF-ULP* patient-reported outcome measure to assess the preferred usage features of upper limb prostheses, *RCRT *refined clothespin relocation test, *BBT* Box and Blocks Test, *SHAP *Southampton Hand Assessment Procedure

- Objective 1. To compare the two prosthetic hands on the categories ‘Activities’, ‘Participation’, and ‘Environmental Factors’, both MHP and SHP users completed questionnaires/scales (between-group comparison). The MHP users completed the set of questionnaires/scales at T1 (Fig. 1). The SHP group completed the same questionnaires/scales, but these were sent to them by post. If any data was missing in the returned surveys, participants were contacted by phone to request missing items.
- Objective 2. To compare the two prosthetic hands on the categories ‘Body Function’ and ‘Activities’, the MHP group executed the same physical measurements on two separate occasions (Fig. 1; T1 and T2). During one measurement participants wore the SHP, provided by the researchers as a loaner if necessary, and during the other measurement, they wore their own MHP (within-group comparison). Since the MHP is controlled with the same two electrodes as the SHP, we assumed that MHP users were also able to control an SHP. To adjust to the SHP, all MHP users were asked to wear the SHP for one week in their home situation before the measurement with the SHP. Which prosthetic hand was used at which measurement was determined with blocked randomization; half of the participants used the SHP at T1, while the other half used the MHP at T1. At T2 the alternative prosthetic hand was used. During the measurements, the participants were instructed to indicate if and when they needed a break to prevent fatigue, as this could negatively influence the data.

## Materials

### Body function

To determine joint coordination, we used an Xsens Inertial & Magnetic Measurement System [22]. The MTw™ sensors (MVN Awinda, Xsens Technologies, Netherlands) were placed on the head, pelvis, sternum, latero-distally on the humerus of both arms, both wrists and hands, and on both scapulae [22]. The sampling frequency was 60 Hz. Before the start of each test (see below), the system was calibrated using the ‘N-pose + walk’ as recommended in the MVN manual [22]. The MVN system automatically calculates every joint angle of the upper extremity. However, for this research we were only interested in the following joint angles: elbow flexion/extension, shoulder flexion/extension/abduction/adduction/internal/external rotation (all on the prosthetic side), trunk flexion/extension/axial, and lateral bending.

Each trial was timed and recorded on video (Sony HDR-CX240E) to visually check the Xsens data and used movement strategies.

The refined clothespin relocation test (RCRT) consists of two tasks, RCRT up and RCRT down [23, 24]. During the RCRT up, participants were asked to pick up three clothespins from a horizontal rod with their prosthetic hand and to place these on the designated spot on a vertical rod (upward direction). In the RCRT down, these three clothespins had to be transferred back from the vertical rod to the horizontal rod (downward direction). A visual aid was placed on the table next to the pinch exerciser to guide the participants to place the correct clothespin at the designated location. Both the upward and downward directions were performed five times. The joint angles in the arm and trunk during task performance were examined to gauge joint angle coordination.

The Tray-test was implemented in the protocol because this test encourages the user to switch to different grips, move the affected arm through different orientations, and perform unimanual as well as bimanual upper limb movements. During the Tray-test participants were asked to pick up a cylinder, which laid horizontally on the top shelf, with their prosthetic hand and to diagonally move the cylinder down, placing it vertically on the bottom shelf [25]. Then, participants were asked to pick up a tray from the bottom shelf with both hands and place it on the upper shelf. The trial ended when the participant was standing in the starting position again after completing the task. The Tray-test was executed ten times.

The outcome measures for the RCRT and Tray-test were completion time, range of motion (RoM), kinematic variability, and kinematic repeatability (see section Data analysis).

### Activities

Three commonly used measures were assessed to examine the execution of tasks and activities of daily living (ADLs): SHAP, BBT, and OPUS-UEFS. The SHAP contains 26 tasks: 12 abstract object tasks and 14 tasks of daily living [26]. The time needed to complete each task was self-timed by the participants. The linear index of function for the prehensile patterns (LIF_pp_) and its weighted version (W-LIF) were calculated [27]. Both scores range from 0 to 100, with higher scores representing better prosthetic hand function [27]. The psychometric properties of the SHAP were tested in ULP users, which supported the internal, construct, concurrent, and discriminant validity [28]. However, also large floor effects and issues with structural validity were identified [28]. Resnik et al. recently developed a new score calculation that minimizes the floor effects: the prosthesis index of functionality (P-IOF) [28], and therefore the P-IOF calculation was added to this study. Even though the SHAP has not been fully validated in persons with ULA, it is a frequently used test in literature [6, 8, 10, 29–31].

The BBT measures the participants’ gross manual dexterity [32]. The participants transport small square wooden blocks from one side of a box over a partition to the other side. The maximum number of blocks transported in 60 s is taken as the score [32]. The test–retest reliability is excellent [33]. Furthermore, differences in scores were found across levels of amputation and amount of prosthesis training, which supports the validity for persons with ULA [33–36]. Note that the starting position of the prosthetic hand for the SHAP and BBT was the closed tripod grip, or when this grip was not set at the MHP the closed tip grip.

Last, we assessed the OPUS-UEFS, which is a self-report survey to determine the ease of execution of ADLs [37, 38]. The 19-item version, which demonstrated good internal construct validity and reliability, was utilized since this version was tested in persons with ULA [11, 37]. The survey was translated by the National Working Group Amputation and Prosthetics of the Arm (WAP-A) of the Dutch Society of Rehabilitation Medicine [39, 40]. Scores range from 0 to 57, with higher scores representing easier execution of ADLs [37, 38].

### Participation

Three self-reported surveys were filled out to investigate the participants’ involvement in life situations: TAPES-upper, EQ-5D-5L, and RAND-36. The psychosocial and prosthesis satisfaction subscales of the TAPES-upper, which were translated into Dutch by the WAP-A, were used to measure the user’s adaptation to upper limb amputation and prosthesis use [41, 42]^.^ The scores were divided into a single prosthesis satisfaction subscale (range: 9–45) and four psychosocial subscales: general adjustment (range: 3–15), social adjustment (range: 4–20), adjustment to limitation (range: 5–25), and optimal adjustment (range: 2–10). Higher scores on the subscales are indicative of satisfaction with prosthesis and psychosocial adjustment to having upper limb amputation and an artificial limb. The TAPES-upper has shown a high internal consistency [41, 43]. Although the prosthesis satisfaction subscale might be classified as an ‘environmental factor’ within the ICF-model, we report it here with the other TAPES subscales.

To assess the health-related quality of life (HRQoL), the Dutch versions of the EQ-5D-5L and RAND-36 were used [44–46]. Although the HRQoL includes multiple categories of the ICF-model [47], we decided to report it here. The EQ-5D-5L consists of two parts. The first part contains five questions regarding mobility, self-care, usual activities, pain, and anxiety/depression. The answers to those five questions describe the overall current health status of a person and is linked to the Dutch scoring algorithm, which generates a single value that expresses the current health status of an individual (range: -0.446 to 1; higher scores indicate better HRQoL) [48]. The second part consists of a visual analogue scale (VAS) on which participants rate their perceived health on a scale of 0 to 100 (higher scores indicate better perceived health). The EQ-5D-5L has been validated in several patient populations [45, 49], however, not specifically for persons with ULA. Additionally, the RAND-36, which was validated for the Dutch population [46] and demonstrated good reliability in a Dutch post-rehabilitation population [50], was used. The RAND-36 consists of 36 items in nine subscales: physical functioning, social functioning, role limitations (physical problem), role limitations (emotional problem), mental health, vitality, pain, general health perception, and health change. The score of each subscale was transformed to a 0–100 scale, in which higher scores indicate a better health state [46].

### Environmental factors

Multiple self-report surveys were used to assess the users’ experience with the MHP or SHP. The D-QUEST evaluates user satisfaction with assistive technology devices [51, 52]. The survey contains 12 questions: eight about the device and four about services. Three scores were calculated (range 1–5; higher scores indicate higher satisfaction): device score, service score, and total score. Good validity and reliability of the D-QUEST have been reported [51].

To determine to what extent the features of the MHP and SHP match with the items that were considered most important by the participating ULP users, a special ePROM to assess the preferred usage features of ULPs (PUF-ULP) was used [19]. The PUF-ULP is an online measure with interactive routines that runs on smartphones and computers in the HealthSnApp application (www.chateau-sante.com/healthsnapp). The content of the PUF-ULP was based on input from 358 Dutch people with ULA and was designed to measure the extent to which an individual’s prosthesis meets the preferred usage features of ULPs [53]. In the PUF-ULP participants were asked to rate their own experiences with their ULP based on the following nine items: wearing comfort; functionality; independence; work, hobby, and household; user-friendliness; life-like appearance; phantom limb pain; overuse complaints; reliability [19]. Each item contained four response levels (e.g., comfortable, fairly comfortable, not very comfortable, uncomfortable). The PUF-ULP is using a special measurement model [54–56] and has been applied to several patient populations [57, 58]. We recently used the PUF-ULP in a Dutch nationwide survey study among ULP users [53]. Based on the PUF-ULP data from 171 survey respondents, weights for each answer level of the items, which represent the range of user experiences with a ULP, were estimated [54]. The answer levels ‘not very user-friendly’ and ‘not user-friendly’ from the item ‘user-friendly’, and answer levels ‘not very reliable’ and ‘not reliable’ from the item ‘reliability’ were merged to facilitate score estimations because respectively only two and one participants rated their ULP experiences in the worst answer levels for those items [53]. We applied the weight estimations calculated in our previous survey study [53] to the population in this study. A total score was calculated by adding up the weights of the provided answer levels from the nine items. The lowest and highest possible scores, if each item was rated on respectively the worst or best level, were − 12.0 and 0.1. However, the scores were transformed, by adding up 12, to scores ranging from 0 to 12.1.

VAS scores of 20 items were used to determine the participants’ opinions about their ULP regarding ease of control, dexterity, donning- and doffing, using a touch screen, pushing/pulling, driving a bike/car, hold/shake hands, natural movements, speed of movements, noise, grip force, size of hand opening, the vulnerability of the prosthesis and glove, maintenance, putting clothes on, required commitment, temperature resistance, physical effort to control, and the healthcare insurance procedure for reimbursement of the prosthesis (range: 0–10; higher score indicate less satisfaction/more effort/etc.). The included items of the VAS scores were based on our previous study, in which we created an extensive overview of items that may be important when selecting a ULP [4]. Items that were already included in the PUF-ULP were excluded from the VAS scores. Items the research team thought were not applicable, not relevant, or overlapped with other items were deleted or adapted. Last, we asked the participants two open questions: (1) what are the main differences between an MHP and SHP for you? (2) why did you choose an MHP or SHP?

### Data analysis

#### Body function

The joint angle data from the MVN-software was exported as Excel files and subsequently imported into MATLAB for further analyses. To this end, customized scripts were written in MATLAB R2018a (MathWorks; Natick, MA, USA).

The start and finish of each trial were determined visually. The start was defined as the first movement seen in one of the shoulder angles: flexion/extension and ab/adduction, as from the starting position elevation of the humerus is needed in all three tasks. The finish was defined as the moment when the shoulder angles flexion/extension and ad/abduction had returned to the starting value. When this moment was unclear, it was approximated with the measured trial completion time. Each trial was time-normalized to 500 steps using a cubic spline, between the start and end of the movement for the kinematic variability and kinematic repeatability. Some trials of the MHP-users showed unaccountable peaks, probably due to gimbal lock, and were excluded from the analyses. For the remaining trials, the RoM of each joint angle was determined, based on the minimum and maximum angle of the raw data for each individual trial. The average RoM for each joint angle was then computed for each participant of all the trials for each test separately.

To assess if joint coordination was similar during repetitive trials, kinematic variability (variability between the movements of the repetitive trials) and kinematic repeatability (similarity of the movements over repetitive trials) were calculated. Literature suggests that less kinematic variability and higher kinematic repeatability in a movement pattern of a prosthesis user may indicate better prosthetic control [16]. The kinematic variability was computed for each test for each joint angle separately by computing the standard deviation over the repetitions of each normalized time point and then calculating the average over all time points. The adjusted coefficient of multiple determination was used to estimate the kinematic repeatability [59]. The coefficient of multiple determination is a statistical measure that assesses the similarity of waveforms. An outcome close to 1 resembles high repeatability, while outcomes close to 0 indicate very low repeatability.

### Statistical analysis

Study data were managed using REDCap data capture tools [60, 61]. The outcome variables were statistically analyzed using IBM SPSS Statistics software, version 23 (IBM Corp., Armonk, N.Y., USA). P-P plots, Kolmogorov–Smirnov, and Shapiro–Wilk tests were used to check if the data was normally distributed before each statistical test was executed. To prevent a type-I error due to multiple testing, the significance level was set at α = 0.01.

#### Within-group comparisons

A paired t-test was used to compare the MHP and SHP conditions within participants (i.e., RCRT up and down, Tray-test, SHAP, BBT). The means of the completion times and standard deviation of the completion times over the trials of the RCRT and Tray-test were calculated and used for the statistical testing. For all paired t-tests, Pearson's *r* was calculated to determine the effect size, with 0.1 < *r* < 0.3 being a small effect, 0.3 < *r* < 0.5 a medium effect and *r* ≥ 0.5 a large effect [62].

For the comparison of the RoM, kinematic variability, and kinematic repeatability between the MHP and SHP, we applied a repeated-measures ANOVA, with factors prosthetic hand (levels: MHP and SHP) and joint angles (levels: trunk flexion/extension, trunk axial bending, trunk lateral bending, shoulder flexion/extension, shoulder internal/external rotation, shoulder abduction/adduction and elbow flexion/extension). With the Mauchly test of sphericity was checked for violation of sphericity. The Greenhouse–Geisser correction was used, when the assumption was not met. The generalized eta squared (η_G_^2^) was calculated to determine the effect size, where 0.02 < η_G_^2^ < 0.13 is considered a small effect, 0.13 < η_G_^2^ 0.26 as a medium effect, and η_G_^2^ ≥ 0.26 as a large effect [63, 64].

#### Between-group comparisons

A chi-squared test with effect size Cramer's V (1 degree of freedom; small: 0.1 < V < 0.3, medium: 0.3 < V < 0.5, large: V ≥ 0.5; 3 degrees of freedom; small: 0.06 < V < 0.17, medium: 0.17 < V < 0.29, large: V ≥ 0.29) was used for the nominal data of the between-group participant characteristics [65]. For the between-group comparisons of continuous data (participants characteristics—ratio data, OPUS-UEFS, TAPES-upper, EQ-5D, RAND-36, D-QUEST, PUF-ULP, VAS-scores), an unpaired t-test was used if the data did not significantly differ from a normal distribution. Homogeneity of variances was checked with Levene’s test. If the data significantly differed from a normal distribution, a Mann–Whitney test was used.

#### Qualitative data

The answers to the two open questions were entered into Atlas.ti software and subsequently categorized by one coder (NK) into four categories: advantages and disadvantages of respectively the MHP and SHP. Illustrative quotes were translated into English.

## Results

### Participants

Fourteen out of the 44 MHP users and 19 out of 49 SHP users who were invited, consented to participate in this study (Fig. 1; Table 1). The physical tests of the MHP group were measured for ten participants at the UMCG and for four participants at Libra Rehabilitation & Audiology. The only significant difference found between the MHP and the SHP groups was that MHP users had fewer years of experience with their current prostheses than the SHP users (Table 1).

**Table 1:** Participant characteristics of MHP and SHP groups

Characteristics	MHP group	SHP group	Test Statistic (t or χ^2^)	df	p-value	Effect size (r or V)
	N(%)/mean ± SD	N(%)/mean ± SD				
N	14 (100)	19 (100)	N/A	N/A	N/A	N/A
Age	48.6 ± 12.4	58.1 ± 15.7	− 1.9	31	0.07	0.3
Sex			0.0	1	0.95	0.0
- Male - Female	9 (64.3) 5 (35.7)	13 (68.4) 6 (31.6)				
Origin of limb absence			0.0	1	0.88	0.0
- Congenital - Acquired	7 (50) 7 (50)	9 (47.4) 10 (52.6)				
Side of limb absence			0.1	1	0.80	0.0
- Left - Right	9 (64.3) 5 (35.7)	13 (68.4) 6 (31.6)				
Wrist type of MHP/SHP			3.7	3	0.30	0.2
- Non-movable wrist - Mechanic wrist - Myoelectric wrist - Not applicable	0 11 (78.6) 3 (21.4) 0	3 (15.8) 13 (68.4) 2 (22.2) 1 (5.3)				
Type of MHP^a^			N/A	N/A	N/A	N/A
- i-Limb^b^ - Bebionic^c^ - Vincent Hand^d^	5 (35.7) 8 (57.1) 1 (7.1)	N/A				
Experience with current prosthesis (years)	3.1 ± 2.8	29.4 ± 19.8	− 5.7	31	0.00*	0.7
Experience with prostheses total (years)	23.7 ± 21.6	37.1 ± 16.3	− 2.0	31	0.05	0.3

^a^See Additional file 1: Table A1 for overview of MHP used per participant

^b^Weight: 432–628 g, speed: 163 mm/s, force: 46–191 N (depending on grip), available grips: 36 [66, 67]

^c^Weight: 402–689 g, speed: 150 mm/s, force: 26–140 N (depending on grip), available grips: 14 [68]

^d^Weight: 399–560 g, speed: 237–288 mm/s, force: 12–44 N (depending on grip), available grips: 15 [69]

*MHP* multi-grip myoelectric hand prosthesis, *SHP* standard myoelectric hand prosthesis, *N* number of participants, *SD* standard deviation

*Statistically significant at p < 0.01 (in bold)

Seven out of the 14 participants (50.0%) of the MHP group had any physical or prosthesis-related particularities at the physical measurements, which are listed in Additional file 1: Table A1. If the SHP group had any physical or prosthesis-related particularities is unknown. Out of the 14 participants, 11 (78.6%) used an SHP before acquiring an MHP (on average for 16.8 ± 14.8 years for 8.8 ± 6.0 h a day). The participants of the MHP group had worn the SHP in the week leading up to the SHP measurement for 3.7 days (± 2.5) and 5.3 h (± 4.7) per day on average.

### Body function

The data from one participant were excluded from the analysis for this category because the participant was unable to complete the RCRT with both MHP and SHP and the Tray-test with the MHP.

#### Completion time

The completion time of the RCRT in the upward direction was significantly slower in the MHP condition compared to the SHP condition (Table 2). However, no statistical difference in the completion time of the RCRT down and Tray-test was found between the MHP and SHP conditions (Table 2). We also found no statistical difference in the standard deviations of the completion time between the MHP and SHP conditions. In Additional file 2: Fig. A1, the completion time (mean, SD) for each trial of each test of each participant can be seen, which shows the variable performance of the two hands within and between participants.

**Table 2:** Within-group comparison of test scores from the MHP group^a^ using the MHP and the SHP

ICF	Measure	MHP condition	SHP condition	Test-statistic (t(df))	p-value	Effect size (r)
		Mean ± SD	Mean ± SD			
B.F.	RCRT up:					
	- Completion time - SD Completion time	23.8 ± 6.8 6.82 ± 4.1	16.3 ± 5.6 2.7 ± 2.9	4.1 (12) 2.8 (12)	0.00* 0.02	0.8 0.6
	RCRT down:					
	- Completion time - SD Completion time	22.4 ± 8.8 6.8 ± 6.3	17.7 ± 7.9 3.3 ± 4.0	1.7 (12) 1.8 (12)	0.12 0.10	0.4 0.5
	Tray-test:					
	- Completion time - SD Completion time	15.6 ± 3.8 4.7 ± 3.1	14.5 ± 4.8 2.9 ± 1.8	1.9 (12) 1.9 (12)	0.30 0.08	0.5 0.5
Act.	SHAP:					
	- LIF_pp_ - W-LIF - P-IOF	46.1 ± 19.8 44.1 ± 20.0 58.9 ± 18.0	53.2 ± 18.7 51.9 ± 18.7 67.4 ± 13.3	− 1.9 (13) − 2.1 (13) − 2.5 (13)	0.08 0.05 0.03	0.5 0.5 0.6
	BBT	17.4 ± 5.5	15.1 ± 7.0	1.6 (13)	0.13	0.4

No statistical differences of the SHAP scores were found between participants who used the Bebionic and i-limb

*MHP* multi-grip myoelectric hand prosthesis, *SHP* standard myoelectric hand prosthesis, *ICF* International Classification of Functioning, Disability, and Health-model, *B.F.* Body Function, *Act. *Activities, *RCRT* refined clothespin relocation test, *SD *standard deviation, *SHAP* Southampton Hand Assessment Procedure, *LIF*_*pp*_ linear index of function for the prehensile patterns, *W-LIF* weighted version of linear index of function for the prehensile patterns, *P-IOF* prosthesis index of functionality; BBT = Box and Blocks Test

^a^N = 14

*Statistically significant at p < 0.01 (in bold)

#### Joint coordination pattern

**Fig. 2:**
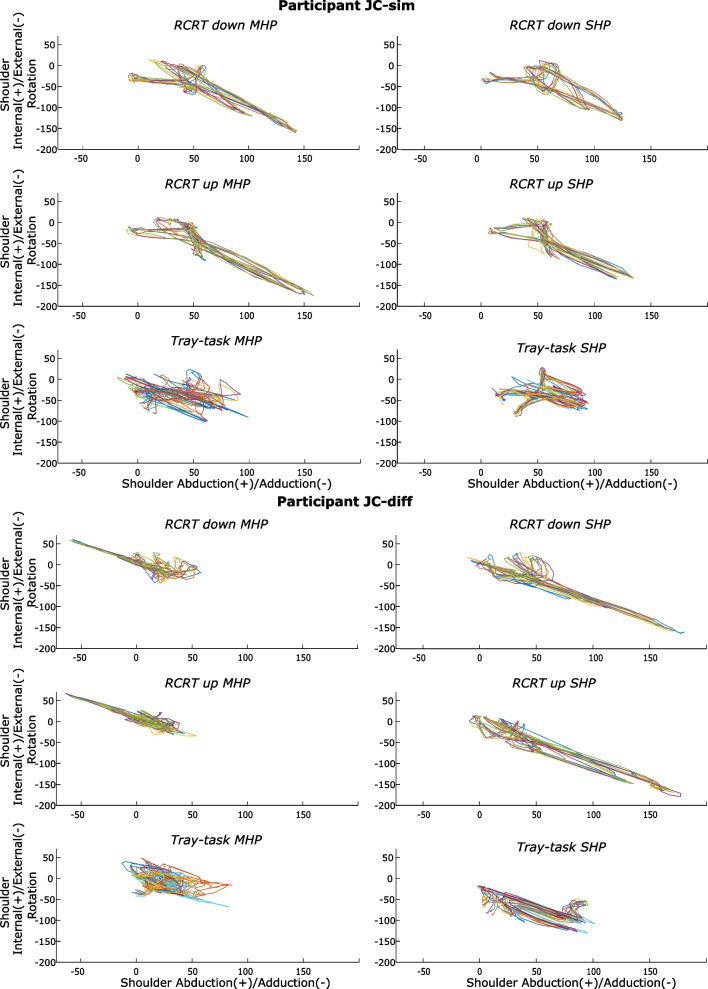
Angle/angle plots of the shoulder internal (+)/external (−) rotation and abduction (+)/adduction (−) from two representative participants. The upper panels show the joint angle coordination of two joint angles of a JC-sim representative for the RCRT up, RCRT down, and Tray-test in the MHP (left panels) and SHP (right panels) conditions. The lower panels show the same for a JC-diff representative. Each colored line is an executed trial*.* During each RCRT trial, three clothespins had to be transferred. The RCRT was executed five times and the Tray-test ten times. *JC-sim* joint coordination similar, *JC-diff* joint coordination different, *RCRT* refined clothespin relocation test, *MHP* multi-grip myoelectric hand prosthesis, *SHP* standard myoelectric hand prosthesis

Perusal of the kinematic profiles of the joint angle data showed that two groups could be distinguished. Both groups will be presented separately. The first group (N = 10, 76.9%), which we call group JC-sim (Joint Coordination-similar), showed qualitatively similar movement patterns in the MHP and SHP conditions. During the RCRT, shoulder external rotation was exploited in combination with shoulder abduction (Fig. 2). No statistical differences were found in RoM of each joint angle between the MHP and SHP for JC-sim for the RCRT up, the RCRT down, and Tray-test (Table 2; Additional file 3: Table A2). The second group (N = 3, 23.1%), called group JC-diff (Joint Coordination-different), showed qualitatively different movement patterns for the RCRT between the MHP and SHP conditions. The JC-diff group exploited shoulder external rotation and abduction while using the SHP, which is the pattern JC-sim showed in both conditions. The JC-diff participants instead exhibited internal rotation and adduction around the shoulder joint to complete the task with the MHP (Fig. 2). These participants in the JC-diff group thus showed a joint coordination where a different range of the shoulder abduction/adduction angle was used for the RCRT up and down in the MHP condition compared to the SHP condition. We should however note that for one of the participants of group JC-diff the difference in movement patterns may be explained by a lower height of the setup of the RCRT in the MHP condition (which we discovered from visually perusing the videotapes after the data analysis). For this participant, the movement patterns of the Tray-test, for which the setup was at the correct heights in both conditions, looked (more) similar in both conditions. This difference in joint coordination during the RCRT could therefore be a consequence of the wrongly adjusted height, as this was not seen in the other two members of the JC-diff group. Due to the small sample, for group JC-diff no statistical tests were performed.

#### Kinematic variability and kinematic repeatability

Kinematic variability and kinematic repeatability for JC-sim did not differ between the MHP and SHP conditions for any of the joint angles for any of the tests (cf. Table 3). The means and standard deviations of the kinematic variability and kinematic repeatability are shown in Additional file 4: Table A3, and Additional file 5: Table A4.

**Table 3:** Test statistics of the repeated measures ANOVA for the RoM, KV, and KR

Interaction effect	Test statistic (F)	df numerator	df denominator	p-value	Effect size (Generalized η^2^)
Prosthetic hand^a^ * Joint angle^b^ RoM					
RCRT up RCRT down Tray-test	0.2 1.9 0.8	1.3 1.6 2.6	11.6 14.2 23.6	0.75 0.20 0.52	0.01 0.03 0.01
Prosthetic hand^a^ * Joint angle^b^ KV					
RCRT up RCRT down Tray-test	0.9 4.0 1.0	1.5 1.7 2.7	13.2 15.3 24.0	0.39 0.05 0.40	0.03 0.09 0.01
Prosthetic hand^a^ * Joint angle^b^ KR					
RCRT up RCRT down Tray-test	1.8 2.0 1.0	3.1 1.9 2.7	28.2 17.4 24.0	0.17 0.16 0.40	0.01 0.01 0.01

The ANOVA was performed separately for each task for the JC-sim group

No statistical differences of the RoM, KV, and KR were found between participants who used the Bebionic and i-limb

^a^Levels: MHP and SHP

^b^Levels: trunk flexion/extension, trunk axial bending, trunk lateral bending, shoulder flexion/extension, shoulder internal/external rotation, shoulder abduction/adduction and elbow flexion/extension

*RoM* range of motion, *KV* kinematic variability, *KR* kinematic repeatability, *RCRT* refined clothespin relocation test, *JC-sim* joint coordination similar, *MHP* multi-grip myoelectric hand prosthesis, *SHP* standard myoelectric hand prosthesis

### Activities

Considering the physical measurements, no differences in prosthetic hand function and gross manual dexterity, measured with respectively the SHAP and BBT, were found between MHP and SHP conditions (Table 2). During the SHAP, one participant (7.1%) did not switch grips on any of the tasks with the MHP, six participants (42.9%) on 1–5 tasks, six participants (42.9%) on 6–10 tasks, and one participant (7.1%) on more than 10 tasks. In the MHP condition, five participants (35.7%) failed one of the tasks of the SHAP, one (7.1%) two tasks, and three (21.4%) four tasks, while in the SHP condition two participants (14.3%) failed one task, four (28.6%) two tasks, and one (7.1%) three tasks. Additionally, six participants (42.9%) exceeded the time limit of 1–5 tasks in the MHP condition, five (35.7%) of 6–10 tasks, and one (7.1%) of more than 10 tasks. In the SHP condition, nine participants (64.3%) exceeded the time limit of 1–5 tasks, one (7.1%) of 6–10 tasks, and two (14.3%) of more than 10 tasks.

Considering the questionnaires/scales, the experienced difficulty in the execution of ADLs, measured with the OPUS-UEFS, did not differ between the MHP and SHP groups (Table 4).

**Table 4:** Between-group comparison of test scores of the MHP group and SHP group

Measure	MHP group (N = 14)	SHP group (N = 19)	Independent T-test	Mann–Whitney test		p-value	Effect size (r)
	Mean ± SD/median (IR)	Mean ± SD/median (IR)	T (df)	U	z		
OPUS-UEFS	47.0 (23.0)	51.0 (15.0)	N/A	104.5	− 1.0	0.30	− 0.2
TAPES-upper							
- Prosthesis satisfaction - General adjustment - Social adjustment - Adjustment to limitation - Optimal adjustment	35.9 ± 5.6 10.5 ± 2.8 15.6 ± 3.9 16.3 ± 4.2 9.0 (4.0)	38.7 ± 5.9 12.7 ± 1.9 16.7 ± 2.2 19.1 ± 4.0 9.0 (2.0)	− 1.5 (31) − 2.7 (31) − 0.9 (19.1) − 2.0 (31) N/A	N/A N/A N/A N/A 129.5	N/A N/A N/A N/A − 0.1	0.19 0.01 0.38 0.06 0.91	0.2 0.4 0.2 0.3 0.0
EQ-5D-5L							
- Health status (utility score) - Perceived health (VAS score) RAND-36 - Physical functioning - Social functioning - Role limitations (physical) - Role limitations (emotional) - Mental health - Vitality - Pain - General health perception - Health change	0.8 (0.2) 83.6 ± 12.8 82.5 (31.3) 93.8 (15.6) 100.0 (75.0) 100.0 (16.7) 80.3 ± 14.6 69.6 ± 13.2 67.4 (22.4) 67.9 ± 19.6 50.0 (31.3)	1.0 (0.1) 83.6 ± 15.6 95.0 (15.0) 100.0 (12.5) 100.0 (0.0) 100.0 (0.0) 84.2 ± 9.1 75.8 ± 12.8 100.0 (0.0) 75.5 ± 20.9 50.0 (0.0)	N/A − 0.0 (31) N/A N/A N/A N/A − 1.0 (31) − 1.3 (31) N/A − 1.1 (31) N/A	35.0 N/A 96.0 112.5 90.0 117.5 N/A N/A 44.0 N/A 96.5	− 3.7 N/A − 1.4 − 0.8 − 2.1 − 0.9 N/A N/A − 3.5 N/A − 1.5	0.00* 0.99 0.17 0.44 0.04 0.48 0.35 0.19 0.00* 0.29 0.10	− 0.6 0.2 − 0.2 − 0.1 − 0.4 − 0.2 0.2 0.2 − 0.6 0.2 − 0.3
D-QUEST							
- Device score - Service score - Total score	3.9 ± 0.5 3.9 ± 0.7 3.9 ± 0.5	4.0 ± 0.4 4.2 ± 0.7 4.1 ± 0.4	− 0.8 (31) − 1.2 (27.0) − 1.1 (31)	N/A N/A N/A	N/A N/A N/A	0.44 0.25 0.28	0.1 0.2 0.2
PUF-ULP	8.9 ± 2.1	10.6 ± 1.3	− 2.8 (31)	N/A	N/A	0.01*	− 0.5
VAS-scores							
- Ease of control - Dexterity - Donning and doffing - Using a touch screen^a^ - Pushing/pulling^b^ - Driving a bike/car^c^ - Hold/shake hands^d^ - Natural movements - Speed of movements - Noise - Grip force - Size of hand opening - Vulnerability of prosthesis - Vulnerability of glove^b^ - Maintenance - Putting clothes on - Required commitment - Temperature resistance - Physical effort to control - The procedure with the healthcare insurance^b^	2.0 (2.0) 2.4 ± 1.8 0.0 (4.0) 10.0 (1.0) 2.7 ± 1.7 2.0 (1.0) 1.9 ± 1.9 3.3 ± 1.9 2.9 ± 2.6 6.0 ± 2.1 4.0 (5.0) 2.0 (4.0) 6.1 ± 2.8 7.2 ± 2.9 4.0 ± 2.8 5.0 ± 2.5 4.9 ± 2.9 6.3 ± 2.3 2.0 (3.0) 4.6 ± 4.0	1.0 (2.0) 2.7 ± 2.2 1.0 (2.0) 9.0 (8.0) 2.8 ± 2.4 0.0 (2.0) 6.6 ± 3.3 4.1 ± 3.0 2.2 ± 2.0 3.1 ± 1.9 1.0 (2.0) 1.0 (4.0) 2.7 ± 1.9 6.2 ± 2.6 2.5 ± 1.4 2.1 ± 1.9 2.6 ± 2.7 4.7 ± 2.9 1.0 (2.0) 2.8 ± 3.4	N/A − 0.4 (31) N/A N/A − 0.1 (30) N/A − 4.1 (19) − 0.8 (31) 0.9 (31) 4.1 (31) N/A N/A 4.2 (31) 1.0 (30) 1.8 (18.7) 3.7 (31) 2.3 (31) 1.7 (31) N/A 1.4 (30)	117.5 N/A 120.5 56.0 N/A 60.0 N/A N/A N/A N/A 60.5 128.0 N/A N/A N/A N/A N/A N/A 62.5 N/A	− 0.6 N/A − 0.5 − 0.7 N/A − 2.0 N/A N/A N/A N/A − 2.7 − 0.2 N/A N/A N/A N/A N/A N/A − 2.7 N/A	0.58 0.72 0.64 0.47 0.90 0.05 0.00* 0.42 0.39 0.00* 0.01* 0.86 0.00* 0.35 0.09 0.00* 0.03 0.11 0.01* 0.16	− 0.1 0.01 − 0.1 − 0.2 0.0 − 0.4 0.7 0.1 0.2 0.6 − 0.5 0.0 0.6 0.2 0.4 0.6 0.4 0.3 − 0.5 1.0

*MHP *multi-grip myoelectric hand prosthesis, *SHP* standard myoelectric hand prosthesis, *OPUS-UEFS* Orthotics and Prosthetics Users’ Survey—The Upper Extremity Functional Status Survey, *TAPES-upper* Trinity Amputation and Prosthesis Experience Scales Upper extremity, *RAND-36* the Research and Development-36, *D-QUEST* Dutch version of the Quebec User Evaluation of Satisfaction with assistive technology, *PUF-ULP* patient-reported outcome measure to assess the preferred usage features of upper limb prostheses, *VAS* visual analogue scales, *SD* standard deviation, *IR* interquartile range

^a^Excluded (n = 9), since activity is not performed with prosthesis

^b^Excluded (N = 1), since activity is not performed with prosthesis

^c^Excluded (N = 3), since activity is not performed with prosthesis

^d^Excluded (N = 12), since activity is not performed with prosthesis

*Statistically significant at p < 0.01 (in bold); N/A = not applicable

### Participation

No differences in the user’s adaptation to upper limb amputation and prosthesis use between the MHP and SHP groups, measured with TAPES-upper, were identified (Table 4). The HRQoL measured with the EQ-5D-5L index scores was significantly lower for the MHP group compared to the SHP group, while the EQ-5D-5L VAS scores did not significantly differ (Table 4). Furthermore, the RAND-36 scores indicated that the MHP group experienced more pain and limitations due to pain compared to the SHP group (Table 4).

### Environmental factors

The D-QUEST results indicated that there was no difference in user satisfaction between the MHP and SHP groups (Table 4). Considering the PUF-ULP results, the match between the users and the preferred usage features of ULPs was lower in the MHP group compared to the SHP group. Additionally, derived from VAS results, the MHP group rated the noise, grip force, vulnerability, difficulties in putting clothes on, and physical effort needed to control regarding their ULP significantly worse compared to the SHP group. Holding and shaking hands was rated significantly better by the MHP group compared to the SHP group (Table 4).

An overview of identified advantages and disadvantages of the SHP and MHP based on the answers to the open questions is provided in Table 5. Frequently mentioned reasons to choose for an SHP were the durability, robustness, grip force, and the all-round usability of the SHP. Furthermore, the SHP group thought the MHP was too vulnerable and difficult to control. Additionally, some SHP users indicated they did not need an MHP since they were satisfied with and/or were already used to their current prosthesis.

‘I have had both types of prosthesis [SHP and MHP]. The flexibility of the bionic hand [MHP] is super cool and really of added value. However, the durability of the glove [of the MHP] is so bad that it does not compensate for the additional options’*—SHP user that had an MHP in the past.*

‘The years of experience with the standard hand have prevented me from applying for a bionic hand [an MHP]’*—SHP user.*

**Table 5:** Overview of the identified advantages and disadvantages of the MHP and SHP

MHP		SHP	
Advantages	Disadvantages	Advantages	Disadvantages
Appearance	Appearance	Appearance	Less embodiment
Better grip	Difficult to control	Durable	Less fine dexterity
Comfort	Expensive	Easy to control	Less functions
Easy to control	Heavy	Fast	Long repair times
Fine dexterity	Less gross dexterity	Functional	More compensatory movements
Keep up with developments	Location for prosthesis training	Higher grip force	Short battery life
Larger hand opening	Moves slow	Less expensive	Small hand opening
Less compensatory movements	Need to turn off the hand to put clothes on	Reliable	Too much grip force (can hurt someone)
More embodiment	Not suitable for activities with water	Robust	
More functions/options	Vulnerable	Suitable for all-round activities	
More natural movements		User friendly	
More safe			
More self-confidence			
Speed is ‘better’			

The table is based on the answers provided to the open questions in the survey

*MHP* multi-grip myoelectric hand prosthesis, *SHP* standard myoelectric hand prosthesis

The MHP group often indicated they chose the MHP because the hand had more options, which made it easier to perform activities, especially more fine motor activities. Furthermore, the MHP group experienced a better grip with the MHP. Whereas some SHP users dislike the appearance of MHPs, an MHP users said she liked the ‘subtle look and thin fingers’ of MHPs. In addition, it seemed that some MHP users liked to go along with the developments in the field.

‘The different grips make it easier to perform different tasks’*—MHP user.*

‘I am a technology enthusiast and always go for the newest things. If a new hand becomes available that can do more than the previous one, I want it too’*—MHP user.*

## Discussion

We evaluated the MHP and SHP on all ICF-categories, ensuring a complete comparison between both types of hands to establish added functionality and user experiences of MHPs. On the ICF-category ‘Body Function’ most users showed a comparable joint angle coordination pattern when using an MHP or SHP. Moreover, in the RCRT and the Tray-test, no significant differences in RoM for any of the joint angles were found between the two hand conditions. Performance on the RCRT up was slower in the MHP condition than in the SHP condition. In the category ‘Activities’, no significant differences were found between the two prosthetic hands. For the category ‘Participation’, the MHP only performed better than the SHP for the VAS-item holding and shaking hands. However, the SHP scored better on the EQ-5D-5L utility score and experienced less pain or limitations due to pain measured with the pain subscale of the RAND-36. Lastly, in the category ‘Environmental Factors’, the SHP group had better experiences on various aspects: PUF-ULP and the VAS items noise, grip force, vulnerability, difficulties in putting on clothes, and physical effort needed to control their ULP. Thus, we found more benefits of the SHP over the MHP in the different ICF-categories. To the authors’ knowledge, this is the first study that examined the MHP and SHP on all the ICF-categories, and also the first one that looked into the differences in joint coordination patterns between the MHP and SHP.

For the category ‘Body Function’, no differences were found in kinematic variability and kinematic repeatability between the prosthetic hand conditions. Previous literature comparing kinematic variability and kinematic repeatability between prosthesis users and able-bodied participants found that kinematic variability was higher for prosthesis users, which could be interpreted as higher motor flexibility in prosthesis users but also as a consequence of unreliable device performance [16]. Our results on kinematic variability would then indicate that the increase of distal Degrees of Freedom from the MHP does not result in an increased number of strategies to execute a task or that the MHP and SHP are equally reliable. We should, however, note that the RCRT test does not require switching grip types when relocating the clothespins, which may explain the non-significant results. The potential advantage of being able to choose from more grip types before execution of the task, such as the lateral grip, also did not lead to less compensatory movements in the RCRT test for MHP users. Even more interesting is that we also did not find any significant differences in kinematic variability nor in kinematic repeatability between the hand conditions in the Tray-test, which was particularly designed to reveal the added value of having multiple grips.

Based on the joint angle coordination strategies, two separate groups of participants could be distinguished: a group of prosthesis users that showed the same coordination pattern with MHP and SHP (N = 10) and a small group of prosthesis users that had a different joint angle coordination pattern between the hands (N = 3). Another study examining the effect of a prosthetic innovation on joint angle coordination found no difference between flexible and static wrists on shoulder angle coordination [29]. Previous studies that investigated compensatory movements in prosthesis users compared to able-bodied participants, found greater shoulder and trunk angles in prosthesis users compared to able-bodied persons, which has been considered to contribute to overuse problems [7, 14–16]. Multiple studies suggested that these increased RoMs could be reduced by using a prosthetic hand capable of multiple grips instead of an SHP [7, 14, 16]. The results of the current study challenge this suggestion since we did not find a decrease in RoM of shoulder and trunk angles in the MHP condition compared to the SHP conditions for the majority of the users. Future research should investigate if the different joint angle coordination strategies shown by some participants will result in fewer overuse problems.

It may be surprising that the MHP, despite the technological innovations, did not result in better outcomes on the ICF-categories compared to the SHP. One reason might be that the type of control (direct control using two electrodes on antagonistic muscles) does not match the many grip options offered by the MHP. To control these grip types, making myoelectric triggers (co-contraction of two antagonistic muscles or making fast twitches, the so-called double pulses) is required. Satisfaction with direct control in combination with an MHP varies between users as some of them experience the switching between grips as time-consuming and mentally demanding [5]. Consequently, switching grips is avoided, limiting the potential added value of the MHP. To overcome this limitation, machine learning control might offer a better fit for the MHP. During machine learning control, multiple electrodes record the activity of the residual muscles, and switching between grips is not needed [70]. In these activation signals, classifier algorithms can recognize patterns in muscle activations. Each pattern then corresponds to a particular grip of the MHP. A preliminary study indicated that machine learning control led to a larger improvement in performance after a home trial compared to direct control [71]. Another possible reason for our results might be a suboptimal design of MHPs. While studies showed that prosthesis users value a robust prosthesis, literature also indicated that MHPs are experienced as fragile [4, 5]. The latter was confirmed in our study, considering the high (worse) VAS score and the qualitative outcomes on vulnerability regarding MHPs. Second, it should be noted that the grip force of MHPs was experienced as low compared to SHPs, which is supported by previous research and our findings (i.e., higher VAS score and slower time on RCRT with MHP due to difficulties with opening clothespins) [5]. While the overall design of MHPs seems insufficient based on previous literature, it is also probable that our findings are the result of differences between the three included MHP types. These MHPs differ in weight, speed (time to close the hand) and the force of the fingers (see footnotes of Table 1). It can, thus, be questioned if the various MHP types can be considered equal. For future research it would therefore be interesting to compare the i-Limb Quantum/Ultra, Bebionic, and VINCENT ULPs with each other, but this would require a new power analysis most likely indicating a larger group of participants. The last reason for our findings might be that the MHP-users in this study were not skilled enough to utilize the full capabilities of the MHP. However, this is difficult to assess since we have not been informed about the quantity and the quality of the prosthesis training the users received. We intended to overcome this limitation by only including participants who had at least six months of experience with their MHP. A recent study showed that there is still a literature gap on how the use of an MHP can be trained most optimally, suggesting that current training methods might not be sufficient [18].

Our results on the categories ‘Activities’ and ‘Participation’ are in line with most literature, that claimed no functional or social benefits of the MHP over the SHP [9, 10, 17, 18, 72]. However, contradictory to previous literature [8, 18], we did not find higher user experience, measured with the PUF-ULP, for the MHP compared to the SHP on the ICF-category ‘Environmental factors’. One explanation might be the difference in study designs. Other studies only made a within-group comparison of the user experiences while in the current study we also compared the outcomes of the questionnaires/scales between a group of MHP and a group of SHP users. Note, the MHP group in our study had significantly less experience with their current prosthesis than the SHP group, which could have influenced our results. Nonetheless, the current study has a bigger and more diverse sample size regarding age and sex in comparison to previous studies [8, 18], which would imply a better representation of the population. Although we did not find a higher user experience, several reasons prosthetic users gave in the open questions for choosing an MHP over an SHP were, apart from its functionality, its appearance, and to keep up with technological developments.

Overall, our findings indicate that the technological innovation of the MHP does not bring the expected functional improvement compared to the SHP. This is of course not the intended aim of implementing innovative technology. One reason might be that user experiences are hardly incorporated when innovating ULPs [73]. To prevent this in the future, a new approach, called co-creation, could be used. This co-creation approach facilitates translating the knowledge gained from research into health care and vice versa [74–76]. During co-creation, multiple stakeholders including the end-users, in this case, the potential prosthesis users themselves, collaborate on a prosthetic research study on each level (i.e., proposal, experiment, analysis, dissemination) [75]. In different fields, such as the development of new rehabilitation exercises for patients with multiple sclerosis, co-creation has already proven to be successful [77, 78]. Adopting a co-creation approach in future research might increase the suitability of new developments to the actual needs of the end-users, which will hopefully result in more effective developments.

This study had some limitations. First, the measurement instruments used in this study were selected because these are state-of-the-art (i.e., RCRT and Tray-test) or have commonly been used in assessing ULP performance (i.e., SHAP and BBT) [23–27]. However, possibly these measurement instruments were not suitable to measure the added value of having multiple grip options. The RCRT has been used before to compare kinematic trajectories of the prosthesis users’ upper limbs but is primarily focused on wrist movements and not on grip switching [23]. The Tray-test was included to encourage prosthesis users to switch between multiple grips, however, casual observations during the experiments suggested that switches between grips were not made often in the current study. A plausible explanation for this finding could be that switching was too complicated, as discussed above. Since the Tray-test was developed recently, improvements to the test might be needed. Lastly, the SHAP was designed to test six different grip patterns [26], however, more than half of the participants hardly switched grips. A possible reason, as suggested by Kyberd and colleagues and mentioned above, might be that the participants were not skilled enough to experience the optimal benefit of the MHP [72]. We should also note that during the execution of the SHAP, we only monitored if the user switched to a different grip, without keeping track which grip was actually used. For future research it might be interesting to investigate which grip types are utilized, not only for the SHAP, but also for other tests. Second, the OPUS-UEFS and TAPES-upper were not officially validated in the Dutch language. Third, the PUF-ULP is a new measurement tool, which has only been used in another yet unpublished study. Although the underlying measurement model is less new and has been used in previous studies [57, 58], future research should investigate the psychometric properties of the PUF-ULP. Fourth, for the within-group comparison, we assumed that MHP users could control an SHP as well, which might not have been the case. However, the fact that the kinematic repeatability did not significantly differ between the prosthetic hands implies that the MHP users had no difficulty controlling the SHP. Fifth, a large number of statistical tests were performed, since we aimed to provide a full overview of the MHP and SHP outcomes in all categories of the ICF model. Therefore, the alpha was corrected to 0.01 to prevent type I error. However, the sample size was calculated based on an α of 0.05. With a lower value for α, more participants would have been needed to find a significant difference. It can be argued that our sample size was limited, but, as the population of MHP users is small, it could be considered large in comparison to previous studies [6, 8, 18].

To conclude, no clear benefits of the MHP over the SHP on all the ICF categories became apparent. The SHP even outperformed the MHP in several outcome measures. As the MHP is expensive to purchase and needs more repairs, prescription of the MHP should be well-considered.

## Supplementary Information

**Additional file 1: Table A1**: MHP types and physical or prosthesis-related particularities of the MHP users at the physical measurements.

**Additional file 2: Fig. A1**: Completion time of the RCRT and the Tray-test with the MHP and SHP for each participant.

**Additional file 3: Table A2**. Descriptives of the RoM for the MHP and SHP for each angle of each task.

**Additional file 4: Table A3**. Descriptives of the KV for the MHP and SHP for each angle of each task.

**Additional file 5: Table A4**. Descriptives of the KR for the MHP and SHP for each angle of each task. The measures are presented separately for JC-sim and JC-diff.

## Data Availability

Data that support the findings of this study are available on DataVerseNL: 10.34894/TDOKCD.

## Abbreviations

ADLs: Activities of daily living
Act.: Activities (i.e., category of ICF-model)
BBT: Box and blocks test
B.F.: Body Function (i.e., category of ICF-model)
D-QUEST: Dutch version of the Quebec User Evaluation of Satisfaction with assistive technology
E.F.: Environmental factors
HRQoL: Health-related quality of life
ICF: International Classification of Functioning, Disability, and Health-model
JC-diff: Joint coordination different
JC-sim: Joint coordination similar
LIF_pp_: Linear index of function for the prehensile patterns
MHP: Multi-grip myoelectric hand prosthesis
N: Number of participants
OPUS-UEFS: Orthotics and Prosthetics Users’ Survey-The Upper Extremity Functional Status Survey
Part.: Participation (i.e., category of ICF-model)
P-IOF: Prosthesis index of functionality
PUF-ULP: Patient-reported outcome measure to assess the preferred usage features of upper limb prostheses
RAND-36: Research and development-36
RCRT: Refined clothespin relocation test
RoM: Range of motion
SD: Standard deviation
SHAP: Southampton hand assessment procedure
SHP: Standard myoelectric hand prosthesis
TAPES-Upper: Trinity amputation and prosthesis experience scales for upper extremity
ULA: Upper limb absence
ULP: Upper limb prosthesis
VAS: Visual analogue scales
W-LIF: Weighted linear index of function for the prehensile patterns
