# Supplementary material for: The multi-grip and standard myoelectric hand prosthesis compared: does the multi-grip hand live up to its promise?
Source: J Neuroeng Rehabil. 2023 Feb 15;20:22. doi: 10.1186/s12984-023-01131-w (PMC9930076; doi:10.1186/s12984-023-01131-w)
Supplement: Supplementary file 1 — Additional file 1: Table A1: MHP types and physical or prosthesis-related particularities of the MHP users at the physical measurements. [file 12984_2023_1131_MOESM1_ESM.pdf]

**Table A.1: MHP types and physical or prosthesis-related particularities of the MHP users at the physical measurements.**

| *Participant* | *MHP type* | *Particulars* |
|---|---|---|
| 1 | i-Limb | N/A |
| 2 | i-Limb | The socket did not fit properly, which resulted in minor malfunctioning in both test conditions |
| 3 | i-Limb | N/A |
| 4 | Bebionic | No wrist function with the MHP but used a passive flexible wrist with the SHP. |
| | | During measurement with SHP, the SHP unintentionally opened and closed sometimes (minor malfunctioning). |
| 5 | BeBionic | The participant is less flexible in elbow and shoulder joints on the prosthetic side. |
| | | The prosthetic hand had a small defect of the thumb in the MHP condition which resulted in a small gap between the thumb and index finger. This influenced some of the grips. |
| | | The participant had an active wrist rotator in both test conditions. However, testers had the idea that the wrist was more commonly used in the MHP condition compared to the SHP condition. |
| 6 | i-Limb | N/A |
| 7 | BeBionic | The participant was wheelchair-bound, he/she had complex regional pain syndrome of the left leg. The tasks that had to be executed standing were done standing on one leg in both test conditions. More and longer breaks were taken to regulate fatigue. |
| 8 | BeBionic | Excluded from data analyses in the ICF category 'Body Function' |
| 9 | i-Limb | N/A |
| 10 | BeBionic | The participant's level of amputation appeared to be at elbow level instead of transradial level. The prosthesis included a mechanical elbow. The elbow was supported with the other arm during some overhand tasks of the measurements in both test conditions. |
| 11 | VINCENT | N/A |
| 12 | BeBionic | Due to a broken clavicle on the prosthetic side in the past, the participant could not lift the arm fully. This caused difficulties when executing the overhand tasks. |
| 13 | BeBionic | N/A |
| 14 | BeBionic | N/A |

*Abbreviations: MHP = multi-grip myoelectric hand prosthesis; N/A = not applicable; SHP = standard myoelectric hand prosthesis; RCRT = refined clothespin relocation test.*
