# Supplementary material for: The multi-grip and standard myoelectric hand prosthesis compared: does the multi-grip hand live up to its promise?
Source: J Neuroeng Rehabil. 2023 Feb 15;20:22. doi: 10.1186/s12984-023-01131-w (PMC9930076; doi:10.1186/s12984-023-01131-w)
Supplement: Supplementary file 2 — Additional file 2: Fig. A1: Completion time of the RCRT and the Tray-test with the MHP and SHP for each participant. [file 12984_2023_1131_MOESM2_ESM.pdf]

Additional file 2

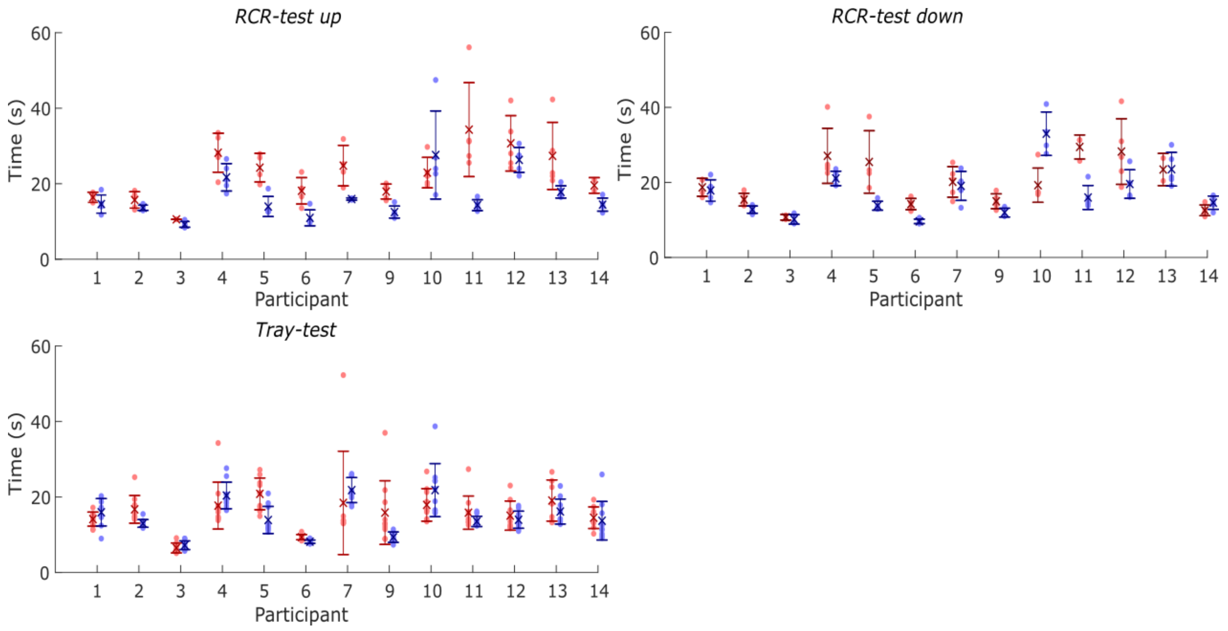

Figure A.1: Completion time of the RCRT and the Tray-test with the MHP and SHP for each participant. The MHP condition is represented in red, while the SHP condition is depicted in blue. During both directions (up and down) of the RCRT, three clothespins had to be transferred, which was completed five times. The Tray-test was executed ten times. The ‘x’ signs represent the mean and the horizontal bars the standard deviation. Each dot represents an individual trial. The time was measured in seconds (s). *Abbreviations: RCRT = refined clothespin relocation test; MHP = multi-grip myoelectric hand prosthesis; SHP = standard myoelectric hand prosthesis.*
