# Supplementary material for: The multi-grip and standard myoelectric hand prosthesis compared: does the multi-grip hand live up to its promise?
Source: J Neuroeng Rehabil. 2023 Feb 15;20:22. doi: 10.1186/s12984-023-01131-w (PMC9930076; doi:10.1186/s12984-023-01131-w)
Supplement: Supplementary file 3 — Additional file 3: Table A2. Descriptives of the RoM for the MHP and SHP for each angle of each task. [file 12984_2023_1131_MOESM3_ESM.pdf]

Table A.2. Descriptives of the RoM for the MHP and SHP for each angle of each task. The measures are presented separately for JC-sim and JC-diff.

		RCRT up		RCRT down		Tray-task	
**RoM**	Group	*MHP*	*SHP*	*MHP*	*SHP*	*MHP*	*SHP*
Elbow Flexion/Extension	*Similar*	58.2 ± 10.1	54.0 ± 24.1	64.3 ± 14.2	58.4 ± 21.6	75.9 ± 26.9	76.4 ± 26.7
	Different	56.00 ± 13.6	70.9 ± 8.7	55.4 ± 16.5	81.0 ± 15.9	70.7 ± 23.9	82.7 ± 36.2
Shoulder Flexion/Extension	*Similar*	82.9 ± 9.4	81.1 ± 6.2	77.6 ± 8.9	75.2 ± 6.9	74.8 ± 8.6	70.3 ± 7.6
	Different	82.3 ± 5.6	84.4 ± 10.9	83.4 ± 8.5	85.2 ± 10.0	71.0 ± 5.3	74.8 ± 7.4
Shoulder Internal/External Rotation	*Similar*	143.2 ± 48.4	130.6 ± 29.1	139.0 ± 28.3	124.4 ± 31.2	73.6 ± 8.1	68.9 ± 15.5
	Different	114.9 ± 59.6	123.6 ± 62.6	97.9 ± 69.1	126.6 ± 74.4	85.4 ± 31.1	78.6 ± 21.0
Shoulder Abduction/Adduction	*Similar*	126.7 ± 48.7	115.8 ± 23.9	117.4 ± 22.0	106.6 ± 23.4	80.7 ± 10.5	79.0 ± 9.3
	Different	109.5 ± 50.8	109.9 ± 54.3	91.4 ± 38.9	111.3 ± 56.4	71.6 ± 24.4	77.6 ± 13.1
Trunk Flexion/Extension	*Similar*	10.0 ± 22.1	2.5 ± 0.9	2.4 ± 0.8	2.4 ± 1.3	3.9 ± 1.4	3.6 ± 1.5
	Different	3.4 ± 1.4	2.9 ± 0.8	3.0 ± 1.4	2.8 ± 1.2	3.8 ± 0.9	3.4 ± 0.4
Trunk Axial Bending	*Similar*	9.4 ± 22.3	2.0 ± 0.4	2.6 ± 0.6	2.5 ± 0.5	3.5 ± 1.0	3.4 ± 0.8
	Different	2.8 ± 0.9	2.2 ± 0.2	3.2 ± 1.4	2.8 ± 0.4	3.4 ± 1.5	3.0 ± 0.8
Trunk Lateral Bending	*Similar*	10.3 ± 22.0	2.8 ± 1.1	3.1 ± 1.2	2.6 ± 1.2	4.6 ± 1.0	4.9 ± 0.9
	Different	3.8 ± 0.5	3.3 ± 1.6	2.7 ± 1.2	2.5 ± 1.3	4.5 ± 1.0	3.6 ± 1.6

Abbreviations: RoM = range of motion; RCRT = refined clothespin relocation test; JC = joint coordination; MHP = multi-grip myoelectric hand prosthesis; SHP = standard myoelectric hand prosthesis
