# Supplementary material for: The multi-grip and standard myoelectric hand prosthesis compared: does the multi-grip hand live up to its promise?
Source: J Neuroeng Rehabil. 2023 Feb 15;20:22. doi: 10.1186/s12984-023-01131-w (PMC9930076; doi:10.1186/s12984-023-01131-w)
Supplement: Supplementary file 4 — Additional file 4: Table A3. Descriptives of the KV for the MHP and SHP for each angle of each task. [file 12984_2023_1131_MOESM4_ESM.pdf]

Table A.3. **Descriptives of the KV for the MHP and SHP for each angle of each task.** The measures are presented separately for JC-sim and JC-diff.

		**RCRT up**		**RCRT down**		**Tray-task**	
**KV**	Group	*MHP*	*SHP*	*MHP*	*SHP*	*MHP*	*SHP*
Elbow Flexion/Extension	*Similar*	10.0 ± 4.1	6.9 ± 3.6	9.2 ± 1.8	7.5 ± 3.4	14.4 ± 2.5	12.8 ± 3.5
	Different	10.3 ± 2.3	8.6 ± 2.3	8.7 ± 1.3	10.3 ± 2.2	11.6 ± 5.2	15.0 ± 8.5
Shoulder Flexion/Extension	*Similar*	11.7 ± 3.8	8.4 ± 3.4	7.9 ± 2.8	6.8 ± 2.1	10.4 ± 1.5	9.2 ± 1.7
	Different	10.0 ± 1.6	6.8 ± 0.6	7.9 ± 1.4	6.9 ± 1.8	10.0 ± 4.1	10.3 ± 1.1
Shoulder Internal/External Rotation	*Similar*	25.0 ± 6.0	19.2 ± 7.7	23.5 ± 6.7	17.1 ± 7.7	13.4 ± 2.9	11.8 ± 1.7
	Different	21.9 ± 15.1	14.3 ± 8.9	18.5 ± 16.1	12.3 ± 3.8	15.2 ± 8.3	12.8 ± 3.0
Shoulder Abduction/Adduction	*Similar*	20.8 ± 3.3	14.9 ± 5.5	15.8 ± 5.2	12.1 ± 5.1	13.9 ± 2.2	13.1 ± 2.6
	Different	18.2 ± 6.8	11.9 ± 5.2	14.1 ± 8.8	9.9 ± 1.7	11.8 ± 6.1	11.9 ± 3.5
Trunk Flexion/Extension	*Similar*	2.4 ± 5.7	0.4 ± 0.2	0.4 ± 0.2	0.4 ± 0.2	0.7 ± 0.3	0.7 ± 0.4
	Different	0.7 ± 0.2	0.4 ± 0.2	0.5 ± 0.2	0.4 ± 0.2	0.7 ± 0.3	0.4 ± 0.1
Trunk Axial Bending	*Similar*	2.2 ± 5.8	0.3 ± 0.1	0.4 ± 0.1	0.3 ± 0.1	0.6 ± 0.1	0.6 ± 0.3
	Different	0.5 ± 0.1	0.3 ± 0.1	0.4 ± 0.1	0.3 ± 0.0	0.5 ± 0.1	0.4 ± 0.0
Trunk Lateral Bending	*Similar*	2.5 ± 5.7	0.5 ± 0.3	0.6 ± 0.3	0.4 ± 0.2	0.7 ± 0.2	0.8 ± 0.2
	Different	0.8 ± 0.1	0.5 ± 0.3	0.5 ± 0.2	0.4 ± 0.2	0.7 ± 0.4	0.4 ± 0.1

Abbreviations: KV = kinematic variability; RCRT = refined clothespin relocation test; JC = joint coordination; MHP = multi-grip myoelectric hand prosthesis; SHP = standard myoelectric hand prosthesis.
