# Supplementary material for: The multi-grip and standard myoelectric hand prosthesis compared: does the multi-grip hand live up to its promise?
Source: J Neuroeng Rehabil. 2023 Feb 15;20:22. doi: 10.1186/s12984-023-01131-w (PMC9930076; doi:10.1186/s12984-023-01131-w)
Supplement: Supplementary file 5 — Additional file 5: Table A4. Descriptives of the KR for the MHP and SHP for each angle of each task. The measures are presented separately for JC-sim and JC-diff. [file 12984_2023_1131_MOESM5_ESM.pdf]

Table A.4. **Descriptives of the KR for the MHP and SHP for each angle of each task.** The measures are presented separately for JC-sim and JC-diff.

		RCRT up		RCRT down		Tray-task	
**KR**	Group	*MHP*	*SHP*	*MHP*	*SHP*	*MHP*	*SHP*
Elbow Flexion/Extension	*Similar*	0.4 ± 0.3	0.5 ± 0.3	0.5 ± 0.2	0.5 ± 0.3	0.5 ± 0.2	0.5 ± 0.2
	Different	0.4 ± 0.2	0.6 ± 0.1	0.4 ± 0.4	0.6 ± 0.1	0.5 ± 0.3	0.6 ± 0.2
Shoulder Flexion/Extension	*Similar*	0.8 ± 0.1	0.9 ± 0.1	0.8 ± 0.1	0.8 ± 0.1	0.7 ± 0.1	0.7 ± 0.2
	Different	0.8 ± 0.2	0.9 ± 0.1	0.9 ± 0.0	0.8 ± 0.2	0.7 ± 0.3	0.7 ± 0.3
Shoulder Internal/External Rotation	*Similar*	1.0 ± 0.0	1.0 ± 0.0	1.0 ± 0.0	1.0 ± 0.0	0.9 ± 0.0	0.9 ± 0.0
	Different	0.9 ± 0.1	1.0 ± 0.0	0.9 ± 0.1	0.9 ± 0.0	0.9 ± 0.1	0.9 ± 0.1
Shoulder Abduction/Adduction	*Similar*	0.8 ± 0.1	0.9 ± 0.1	0.9 ± 0.1	0.9 ± 0.1	0.7 ± 0.1	0.8 ± 0.2
	Different	0.8 ± 0.2	0.9 ± 0.0	0.8 ± 0.1	0.8 ± 0.1	0.7 ± 0.3	0.7 ± 0.3
Trunk Flexion/Extension	*Similar*	0.7 ± 0.3	0.8 ± 0.2	0.7 ± 0.2	0.8 ± 0.2	0.8 ± 0.1	0.8 ± 0.1
	Different	0.7 ± 0.2	0.8 ± 0.2	0.7 ± 0.2	0.8 ± 0.1	0.8 ± 0.1	0.8 ± 0.0
Trunk Axial Bending	*Similar*	0.8 ± 0.2	0.8 ± 0.3	0.8 ± 0.2	0.8 ± 0.3	0.9 ± 0.1	0.9 ± 0.1
	Different	0.8 ± 0.1	0.8 ± 0.2	0.8 ± 0.1	0.8 ± 0.1	0.9 ± 0.1	0.8 ± 0.0
Trunk Lateral Bending	*Similar*	0.8 ± 0.2	0.8 ± 0.3	0.8 ± 0.2	0.8 ± 0.3	0.9 ± 0.1	0.9 ± 0.1
	Different	0.8 ± 0.1	0.8 ± 0.2	0.8 ± 0.1	0.8 ± 0.1	0.9 ± 0.1	0.8 ± 0.0

Abbreviations: KR = kinematic repeatability; RCRT = refined clothespin relocation test; JC = joint coordination; MHP = multi-grip myoelectric hand prosthesis; SHP = standard myoelectric hand prosthesis.
